# Balanced Duality: H_2_O_2_-Based Therapy in Cancer and Its Protective Effects on Non-Malignant Tissues

**DOI:** 10.3390/ijms25168885

**Published:** 2024-08-15

**Authors:** Amira Zaher, Michael S. Petronek, Bryan G. Allen, Kranti A. Mapuskar

**Affiliations:** Department of Radiation Oncology, The University of Iowa, Iowa City, IA 52242, USA; amira-zaher@uiowa.edu (A.Z.); michael-petronek@uiowa.edu (M.S.P.); bryan-allen@uiowa.edu (B.G.A.)

**Keywords:** hydrogen peroxide, H_2_O_2_, signaling, inflammation, antioxidant, prooxidant, ROS, immunity, cancer therapy, radiation, chemotherapy, immunotherapy, normal tissue injury, chemotoxicity, radiation injury, pharmacological ascorbate, superoxide dismutase mimetics

## Abstract

Conventional cancer therapy strategies, although centered around killing tumor cells, often lead to severe side effects on surrounding normal tissues, thus compromising the chronic quality of life in cancer survivors. Hydrogen peroxide (H_2_O_2_) is a secondary signaling molecule that has an array of functions in both tumor and normal cells, including the promotion of cell survival pathways and immune cell modulation in the tumor microenvironment. H_2_O_2_ is a reactive oxygen species (ROS) crucial in cellular homeostasis and signaling (at concentrations maintained under nM levels), with increased steady-state levels in tumors relative to their normal tissue counterparts. Increased steady-state levels of H_2_O_2_ in tumor cells, make them vulnerable to oxidative stress and ultimately, cell death. Recently, H_2_O_2_-producing therapies—namely, pharmacological ascorbate and superoxide dismutase mimetics—have emerged as compelling complementary treatment strategies in cancer. Both pharmacological ascorbate and superoxide dismutase mimetics can generate excess H_2_O_2_ to overwhelm the impaired H_2_O_2_ removal capacity of cancer cells. This review presents an overview of H_2_O_2_ metabolism in the physiological and malignant states, in addition to discussing the anti-tumor and normal tissue-sparing mechanism(s) of, and clinical evidence for, two H_2_O_2_-based therapies, pharmacological ascorbate and superoxide dismutase mimetics.

## 1. Introduction

Hydrogen peroxide (H_2_O_2_) was discovered in 1818 by French chemist Louis Jacques Thénard for its use as a bleaching agent and disinfectant and in the late 19th to early 20th centuries for its antiseptic qualities. Over 200 years later, the knowledge of the nuanced roles of H_2_O_2_ within biological systems makes it one of the most studied molecules in biology for its complex roles in health and disease [1,2,3]. H_2_O_2_ is a reactive oxygen species and a weak acid primarily generated within the mitochondria as a byproduct of aerobic respiration and by some enzymes, including NADPH oxidases (NOXs). Mitochondria generate H_2_O_2_ through mitochondrial superoxide dismutase (SOD2), which catalyzes the removal of superoxide (O_2_^•−^) produced by a leak of electrons from redox centers (complexes I, II, and III) in the electron transport chain (ETC) and yields H_2_O_2_ [4,5]. Therefore, O_2_^•−^-generating NOX enzymes (e.g., NOX2) can generate H_2_O_2_ indirectly through the dismutation of superoxide released by the enzyme. In contrast, NOX4 can directly generate H_2_O_2_ through the activity of their peroxidase-like domain [6,7]. 

The ability of H_2_O_2_ to diffuse across membranes and react with various biomolecules, including DNA, proteins, and lipids, has been shown to disrupt cellular function, leading to cell death (Table 1) [8]. H_2_O_2_ can impair mitochondrial function, leading to decreased ATP synthesis and increased oxidative stress [9]. This in turn creates a bioenergetic deficiency, creating a positive feedback loop that exacerbates cell death, particularly in cancer cells. H_2_O_2_ can also cause damage through mechanisms that include Fenton chemistry, which involves the reaction between H_2_O_2_ and redox-active iron, leading to the formation of hydroxyl radicals that can, in turn, induce site-specific DNA damage, in addition to lipid and protein damage [10]. Additionally, protein modifications caused by H_2_O_2_ lead to redox changes, irreversible oxidation, protein fragmentation, mitochondrial oxidative protein accumulation, and kinase activation that subsequently affect the structural and functional integrity of proteins [11,12,13]. Therefore, H_2_O_2_ levels are tightly regulated through an antioxidant defense machinery composed of antioxidant enzymes, including catalase (CAT), glutathione peroxidase 1 (GPx1), and peroxiredoxins (Prxs) [14,15,16]. Catalase can efficiently remove H_2_O_2_ by directly converting it to oxygen (O_2_) and water (H_2_O) [14,15]. GPx1 utilizes intracellular glutathione (GSH) as a reducing equivalent for converting H_2_O_2_ into H_2_O, whereas Prxs utilize electrons from thioredoxin to reduce H_2_O_2_ to H_2_O [14,16]. Collectively this intricate antioxidant enzyme network regulates the intracellular steady-state levels of H_2_O_2_ to enable its functional utilization and maintain a non-toxic homeostasis. As an endogenous oxidant, H_2_O_2_ is a relatively stable form of reactive oxygen species (ROS) that participates in cellular activities, including proliferation, redox signaling and regulation, antioxidant function, and inflammation [17]. However, disruptions in H_2_O_2_ metabolism have been linked to a plethora of diseases, including diabetes, neurodegenerative disease, cardiovascular disease, and cancer [18,19,20,21]. Despite significant advancements in cancer therapy over the past two decades, a poor prognosis, chemo- and radioresistance, and therapy-associated toxicities persist that warrant the critical need for novel approaches to enhance conventional chemoradiation with a minimal toxicity to normal tissues. In this regard, targeting H_2_O_2_ metabolism has emerged as a promising approach in cancer therapy, due to its multifaceted role in regulating cancer cell proliferation and death. This article discusses the physiological roles of H_2_O_2_, the mechanisms of utilization and metabolism of H_2_O_2_ by cancer cells, the strategies for targeting H_2_O_2_ in cancer, and the role of H_2_O_2_ in mitigating normal tissue toxicities. 

## 2. H_2_O_2_ as a Signaling Molecule

The conventional role of H_2_O_2_ has been synonymous with ROS and oxidative stress (the pathological accumulation of ROS); over the years, research has highlighted its function as a signaling molecule in several physiological processes. This pleiotropic nature of H_2_O_2_ underlines its dichotomy as a damaging agent and a signaling molecule. 

The relative stability, tight regulation, and protein oxidation capacity of H_2_O_2_ make it an ideal second messenger for normal cell function and survival. As a second messenger, H_2_O_2_ alters protein activation via the reversible oxidation of cysteine residues on proteins into sulfonic acid or disulfide bonds with neighboring cysteines [22]. This section will highlight examples of how H_2_O_2_ as a signaling molecule can impact cell growth and survival, transcription, ion channel function, and immune modulation. A recent large-scale analysis revealed over 1000 cysteine-containing proteins potentially oxidizable by H_2_O_2_ [23]. This was consistent with previous findings indicating the involvement of H_2_O_2_ in the reversible oxidation of a variety of kinases, phosphatases, serum proteins, redox-regulating proteins, transcription factors, and oxygen carriers [22,23]. PTEN, a tumor suppressor phosphatase, is oxidized by H_2_O_2_ at Cys-124 in the active site. This leads to a disulfide bond formation with Cys-72 and the subsequent inhibition of the protein, thus promoting cell growth [24]. SH2 domain-containing phosphatases or hematopoietic cell phosphatases (SHPs), known as negative regulators of growth and proliferation, are also regulated by H_2_O_2_, where SHP-1 is oxidized at Cys-455 and SHP-2 at Cys-459, resulting in SHP inactivation and increased proliferation [25,26]. H_2_O_2_ can also promote proliferation by acting on kinases such as EGFR, where Cys-797 is oxidized by H_2_O_2_, resulting in sulfenylation of the residue and increased kinase activity and proliferation [27]. Conversely, H_2_O_2_ can suppress growth and proliferation, such as in the case of Akt2, where H_2_O_2_ acts as a negative regulator to reduce Akt2 activity by oxidizing Cys-124 [28]. These examples demonstrate the crucial role of H_2_O_2_ in cell survival, engaging the audience in the intricate mechanisms that regulate cell fates. 

Some ion channels are also regulated by H_2_O_2_. As a mediator of cellular homeostasis, H_2_O_2_ contributes to vascular tone through large conductance calcium-activated potassium (BK_Ca_) and voltage-dependent potassium (K_V_). The pore-forming α-subunit of the K_v_ channel, Kv1.5, is essential for regulating contractility, motility, proliferation, and adhesion in smooth muscles, brain cells, and disease states, including cancer and atrial fibrillation (AF) [29,30,31,32,33]. The H_2_O_2_-mediated post-translational modification of Kv1.5, via sulfonic acid formation at Cys-581, leads to internalization of the channel and a significant reduction of potassium-dependent polarization [34]. Other ion-sensitive proteins regulated by H_2_O_2_ include the neuronal calcium-binding proteins calbindin-D28k [35,36]. Calbindin D-28k contains three redox-sensitive cysteines that are thought to be targets of H_2_O_2_: Cys-187, 219, and 257 [37]. The oxidation of these residues reduces the calcium-binding affinity of calbindin D-28k and potentially leads to a reduction in calcium buffering and transport capacity [35,37]. 

When it comes to transcription factors, Nrf2 and NF-kB are noteworthy examples of H_2_O_2_ signaling regulating transcription. Despite being a reactive oxygen species, H_2_O_2_ plays a pivotal role in maintaining the balance between oxidative eustress (the physiologically beneficial well-tolerated accumulation of ROS) and oxidative stress. H_2_O_2_ promotes both the nuclear translocation and de novo synthesis of the transcription factor Nrf2 in a concentration-dependent manner, stimulating the transcription of several antioxidant genes, including CAT, PRDX, SOD, and GPX [38,39]. NF-kB is another transcription factor regulated by H_2_O_2._ H_2_O_2_ can regulate inflammation through its signaling action, which activates the transcription factor NF-kB by promoting the phosphorylation of IκBα, leading to NF-kB nuclear translocation and DNA binding that induces the expression of a plethora of genes associated with inflammation, such as IFNG, IL6, and CXCL11 [40,41]. In contrast, H_2_O_2_ can oxidize Cys-62 on the P50 subunit of NF-kB, thereby suppressing its DNA binding and the subsequent transcriptional events and associated inflammatory responses [42]. The effects of H_2_O_2_, therefore, may influence multiple cell populations, including immune cell populations such as neutrophils and macrophages.

Beyond NF-kB, H_2_O_2_ acts as a pro-inflammatory first messenger of cell signaling, H_2_O_2_ functionally contributes to inflammation and immune modulation. H_2_O_2_ is a chemoattractant for neutrophils via the Ca^2+^ permeable, transient receptor potential melastatin 2 (TRPM2) ion channel in vitro and in vivo, wherein increased concentrations of H_2_O_2_ lead to overactivation of TRPM2, flooding the cell with Ca^2+^ and halting neutrophil movement [43]. Conversely, an anti-inflammatory role for H_2_O_2_ has been noted in neutrophil activation in acute lung injury [44]. Zmijewski et al. showed an increase in the levels of H_2_O_2_ in acatalasemic (low levels of catalase) neutrophils and in neutrophils exposed to aminotriazole (AT) [44]. Furthermore, decreased IκB-α degradation, NF-κB nuclear accumulation, proinflammatory cytokines TNF-α, and macrophage inhibitory protein (MIP)-2 were also observed in acatalasemic neutrophils and aminotriazole-exposed neutrophils, along with a reduced severity of LPS-induced acute lung injury, indicating an anti-inflammatory role for H_2_O_2_ [44]. In addition to acting as a chemoattractant, H_2_O_2_ was also shown to aid in T cell migration upon chemokine signaling, whereby H_2_O_2_ enters T cells through aquaporin-3, activating Cdc42, thereby promoting migration [45,46]. Moreover, H_2_O_2_ was shown to activate NF-kB in monocytes/macrophages during their oxidative burst [47,48]. In addition to macrophage activation, H_2_O_2_ has been reported to regulate M1/M2 macrophage polarization in a concentration-dependent manner, where high concentrations promote M1 macrophages and lower concentrations promote M2 polarization [49]. These observations highlight the various mechanisms by which H_2_O_2_ may alter immune cell recruitment, migration, and activation. 

The role of H_2_O_2_ in signaling, inflammation, the fine-tuning of cell function, and maintaining redox homeostasis makes it an essential mediator of cellular processes and, as a result, an attractive therapeutic target for various pathologies, including cancer. 

## 3. H_2_O_2_ in Cancer: A Sliding Scale

**Figure 1:**
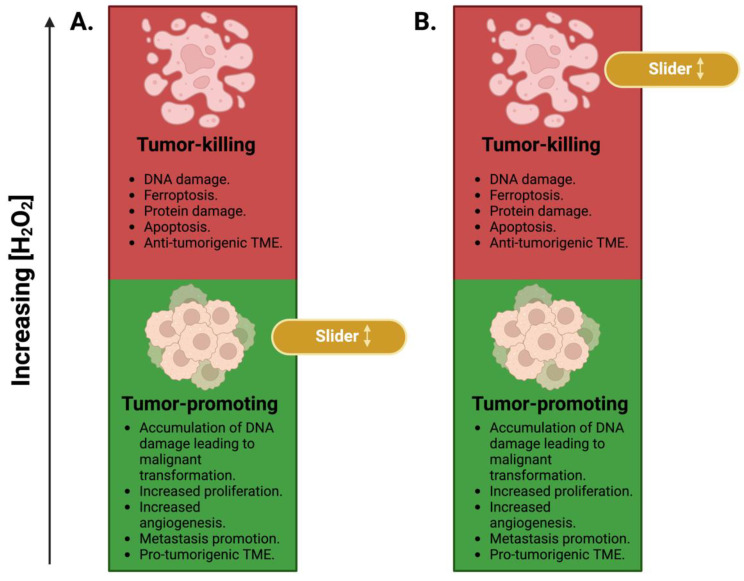
The sliding scale of the concentration-dependent effects of intracellular H_2_O_2_ on cancer cells. (**A**) The tumor-promoting region of H_2_O_2_ in cancer and its associated effects. (**B**) The tumor-killing region of H_2_O_2_ in cancer and its related effects.

Under physiological conditions, intercellular concentrations of H_2_O_2_ are tightly regulated by antioxidants (under nM concentrations) [50,51,52,53]. Cancer cells, however, maintain elevated concentrations of H_2_O_2_ [54,55,56,57,58]. These increased concentrations of H_2_O_2_ illustrate the ability of cancer cells to manipulate normal cellular processes, maximize their proliferative capacity, and exploit the role of H_2_O_2_ as a signaling molecule to promote metastasis. Moreover, due to its role in proliferation and inflammatory signaling, H_2_O_2_ is a central metabolite in cancer biology. H_2_O_2_ has been shown to induce malignant transformation, likely due to its ability to induce DNA damage [10,59,60]. The metabolism of H_2_O_2_ in cancer can be described as a sliding scale with the slider moving across two regions: a tumor-promoting region and a tumor-killing region (Figure 1). The tumor-promoting region consists of redox characteristics such as H_2_O_2_ dependency to promote proliferation (Figure 1A), whereas the tumor-killing region can be characterized by even higher H_2_O_2_ concentrations that surpass their cytotoxic threshold, which overwhelm the acquired ROS-related adaptations in the tumor, leading to DNA, lipid, and protein damage that triggers cell death machinery in the cell [61,62,63] (Figure 1B). 

In the tumor-promoting region, NOX enzymes have been shown to contribute significantly to the elevated intracellular levels of H_2_O_2_ [64,65]. Data from the cancer genome atlas (TCGA) have shown that cancers of epithelial origin, including skin, lung, prostate, liver, stomach, brain, head and neck, and breast, overexpress one or more NOX enzymes, particularly NOX4 and DUOX1/2 [65]. Using NOX4 siRNA and NOX4 inhibitors, Yamaura et al. showed that NOX4-generated ROS/H_2_O_2_ is required for tumor progression in the melanoma transformation phenotype by promoting cell cycle progression through phase G2 [66]. In non-small-cell lung cancer (NSCLC), NOX1-generated H_2_O_2_ promotes metastasis by activating TLR-4 signaling independently of mitochondrial ROS [67]. In glioblastoma multiforme (GBM), NOX4-derived ROS have been shown to promote proliferation and radioresistance [68,69]. Furthermore, NOX4 overexpression corresponded with poor clinical outcomes in human colorectal cancer patients and increased proliferation and invasion in vitro [70]. These studies highlight the significance of NOX enzymes as sources of ROS/H_2_O_2_ in tumor promotion. 

In addition to NOX hyperactivation, the mitochondrial dysfunction associated with rapid proliferation further exacerbates H_2_O_2_ accumulation in cancers [71,72]. This increase in steady-state levels of H_2_O_2_ can promote the tumor landscape, including proliferation, angiogenesis, and metastasis, and facilitate alterations in the tumor microenvironment (TME). H_2_O_2_, as a signaling molecule, can promote proliferation by acting on tumor suppressors or tumor promoters through the previously discussed cysteine oxidations, and cancer cells exploit this feature to promote proliferation. For example, in lung cancer, aquaporin-3-mediated H_2_O_2_ intake resulted in the inactivation of the tumor suppressor, PTEN, resulting in tumor progression [73]. H_2_O_2_ was found to promote gastric cancer cell proliferation by activating EGFR, one of the targets for H_2_O_2_-mediated cysteine oxidation, as previously discussed [27,74]. Similarly, H_2_O_2_ induced AP-1 activation in prostate cancer cells, increasing proliferation [75]. Moreover, breast and lung cancer cells demonstrated a concentration-dependent increase in proliferation when exposed to H_2_O_2_ [76]. Jerónimo et al. showed that H_2_O_2_ regulates angiogenesis in multiple tumor cells, including breast, colon, lung, and brain cancers [77]. These data were in line with previous findings by Xia et al., reporting that H_2_O_2_ was required for angiogenesis in ovarian cancer [78]. H_2_O_2_ has been shown to act as a tumor-promoting agent in metastasis. In pancreatic tumors, H_2_O_2_ promotes epithelial-to-mesenchymal transition and invasion by acting on EGFR, NF-kB, and ERK, an effect that was suppressed by catalase overexpression [79,80,81]. Similarly, the targeted delivery of catalase inhibited H_2_O_2_-mediated metastasis in a murine model of hepatic metastasis [82]. This metastasis-promoting effect of H_2_O_2_ was also observed by Stemberger et al. in breast cancer, Nelson et al. in sarcoma, and Polytarchou et al. in prostate cancer [75,83,84].

In addition to promoting proliferation, angiogenesis, and metastasis, H_2_O_2_ is believed to alter the TME to promote pro-tumorigenic inflammation. Martinez-Outschoorn et al. showed that hydrogen peroxide from tumor cells acts as a “fertilizer” in the TME by secreting H_2_O_2_ to target neighboring cancer-associated fibroblasts (CAFs), to propagate oxidative stress that results in CAF damage and “nutrient transfer” from CAFs to tumor cells, thus supporting tumor growth [85]. The oxidative stress and inflammation due to cancer-associated H_2_O_2_ can also impact the immune cell populations in the TME. ROS, including H_2_O_2_, have been shown to suppress anti-tumor T cell responses by promoting the generation of myeloid-derived suppressor cells, which in turn suppress T cell activation and proliferation [86]. A similar effect was observed with tumor-associated macrophages, which secrete ROS to suppress anti-tumor T cell function by signaling to activate immunosuppressive T regulatory cells, leading to increased tumor growth [87]. Furthermore, high levels of H_2_O_2_ and other ROS can promote T cell exhaustion, as the function of the T cell exhaustion PD-1 protein has been shown to correlate with ROS [88]. Taken together, it can be concluded that increased H_2_O_2_ concentrations in tumors can serve a tumor-promoting function by targeting pathways that promote proliferation, angiogenesis, and metastasis and by potentially targeting other cell populations in the TME. 

Cancer cells are thought to acquire adaptations that accommodate their elevated steady-state levels of H_2_O_2_. These adaptations can vary among malignancies and often involve alterations to antioxidant transcription factors (e.g., Nrf2), NADPH, and iron metabolism to maintain that high level of H_2_O_2_ [21,89,90]. However, with H_2_O_2_ concentrations increased over the cytotoxic threshold, the slider moves toward the tumor-killing region (Figure 1B), wherein cancer cells fail to remove the excess H_2_O_2_, resulting in a higher propensity for DNA damage, apoptosis, ferroptosis, and protein damage. This principle is based on studies that previously demonstrated that most cancer cells have a significantly reduced capacity to remove H_2_O_2_ compared to their non-malignant counterparts [91,92]. An endogenous increase in H_2_O_2_ and a decreased H_2_O_2_ removal capacity leads to an impairment in eliminating excess H_2_O_2_ above their threshold, leading to cancer cell death. This presents H_2_O_2_ metabolism as a therapeutic vulnerability that can be exploited in various pathologies. The rationale for targeting H_2_O_2_ is based on the premise that increasing H_2_O_2_ levels in the tumor and its microenvironment (TME) can shift the balance toward cancer cell death. This strategy promotes DNA and protein damage, induces apoptosis and ferroptosis, and fosters an anti-tumorigenic TME, thereby enhancing the overall efficacy of cancer therapy. This principle led to numerous discoveries and successful clinical trials using H_2_O_2_-generating agents, such as pharmacological ascorbate and superoxide dismutase mimetics. 

## 4. Targeting H_2_O_2_ in Cancer: Enhancing Conventional Therapy and Minimizing Its Normal Tissue Toxicities

Numerous findings indicate the significance of H_2_O_2_ in cancer growth, angiogenesis, and metastasis. Thus, consequent efforts have been made to develop therapies targeting H_2_O_2_ in cancer. This section focuses on two H_2_O_2_-based cancer therapeutic modalities, pharmacological ascorbate (gram doses delivered intravenously reaching plasma concentrations ≥ 20 mM; P-AscH^−^), and superoxide dismutase (SOD) mimetics, in the context of the balanced duality of these agents to potentially protect non-malignant tissues from therapy-induced toxicities. 

### 4.1. Pharmacological Ascorbate

Ascorbate (ascorbic acid or Vitamin C) is an essential dietary vitamin for multiple cellular processes such as collagen biosynthesis, antioxidant defense, epigenetic regulation, and immune function [93]. Ascorbate can undergo autooxidation or metal-catalyzed oxidation, resulting in H_2_O_2_ formation. However, metal-catalyzed ascorbate oxidation is thermodynamically favorable at a physiological pH of 8.5. Dietary vitamin C intake accomplishes physiological plasma concentrations of ≈0.8 mM, tightly regulated through urinary excretion [94,95,96,97]. However, when administered intravenously, ascorbate demonstrates exceptional pharmacokinetics that circumvents physiological excretion and allows plasma concentrations to reach upwards of 20 mM [95,96,98]. This pharmacokinetics is precipitated in the concept of P-AscH^−^, which indicates high doses of intravenous vitamin C, yielding supraphysiological plasma concentrations [95]. P-AscH^−^ became one of the emerging strategies for cancer therapy concurrent with conventional chemoradiation, particularly following the discoveries of Mark Levine and his collaborators in pre-clinical settings, which demonstrated that P-AscH^−^ can selectively induce cancer cell killing, while sparing non-malignant cells in an H_2_O_2_-dependent manner [61,99,100,101]. Studies by Levine et al. showed that P-AscH^−^ delivers H_2_O_2_ to the tumor cells and participates in Fenton reactions with freely chelatable, redox-active intracellular iron, leading to oxidative damage [61,99,100]. Simultaneously, in non-malignant cells, the pro-oxidant role of P-AscH^−^ is dampened by the increased metabolism of H_2_O_2_ [91,102,103]. Additionally, a tightly regulated labile iron pool in non-malignant cells alleviates the pro-oxidant effects of P-AscH^−^, thereby allowing it to function as an antioxidant [102,103]. This conclusion was based on two studies by Chen et al. where P-AscH^−^ demonstrated selective cytotoxicity in human lymphoma and breast cancer and murine colon, lung, melanoma, and kidney cancers [61]. Conversely, minimal toxicity was observed in normal breast cells, fibroblasts, and blood cells [61]. These findings were later confirmed in another study, which showed that cancer cells are less efficient at metabolizing H_2_O_2_ than non-cancerous cells, using pancreatic, lung, liver, intestine, and skin cancer cells and normal cells from the corresponding tissues [91]. 

The cytotoxic effects of P-AscH^−^ on cancer cells were reproduced in vitro in numerous studies on brain, colon, pancreatic, ovarian, prostate, breast, and other cancer cells [104,105,106,107,108,109]. Consistently, P-AscH^−^ demonstrated toxicity in murine models of various cancers, along with a significant enhancement of chemotherapy and radiation. Xenograft models of ovarian and pancreatic cancers that utilized P-AscH^−^ as a single agent demonstrated that daily administration of P-AscH^−^ showed a significant reduction in tumor burden by up to 53% [99]. Similarly, in a syngeneic hepatocarcinoma model, P-AscH^−^ as a single agent significantly reduced tumor growth by 40% [110]. Single-agent P-AscH^−^ also showed a significant reduction in tumor burden and metastasis in a syngeneic model of hormone-refractory prostate cancer [101]. The findings of these studies generated interest in the clinical utilization of P-AscH^−^, concurrently with conventional chemoradiation. P-AscH^−^, combined with chemotherapy and/or radiation, significantly increased therapeutic responses in multiple in vivo tumor models. When combined with paclitaxel, carboplatin, and radiation in gastric cancer, P-AscH significantly increased overall survival and reduced metastases [111]. Another study on gastric cancer consistently showed that P-AscH^−^ significantly enhanced the efficacy of oxaliplatin and irinotecan [109]. Pancreatic cancers, known for their poor clinical outcomes, demonstrated striking sensitivity to concurrent P-AscH^−^ in vivo. Overall survival significantly increased when P-AscH^−^ was combined with radiation or FOLFIRINOX [112,113]. In ovarian cancer, P-AscH^−^ significantly improved the efficacy of the PARP inhibitor Olaparib [114]. Concurrently with radiation and ATM inhibition, P-AscH^−^ significantly increased overall survival in a syngeneic and a xenograft model of colorectal cancer [115]. Consistently, murine models of non-small-cell lung cancer (NSCLC) and glioblastoma (GBM) showed that P-AscH^−^ is synergistic with radiation combined with carboplatin in NSCLC and with radiation and temozolomide in glioblastoma (GBM) [103]. 

Encouraging advances in preclinical studies on the efficacy of P-AscH proved to be a segue into clinical studies, paving the way for multiple clinical trials that combined P-AscH^−^ with conventional chemoradiation to enhance therapeutic efficacy (summarized in Table 2). In GBM, patients in a phase 2 clinical trial who received P-AscH^−^, in addition to radiation and temozolomide, demonstrated a remarkable increase in median overall survival, from 14.6 months in historical controls to 19.6 months in the trial [116]. In ovarian cancer patients, a phase 1/2a trial reported that patients receiving P-AscH^−^ concurrently with carboplatin and paclitaxel demonstrated an increase in progression-free survival at 25.5 months, compared to 16.75 months in patients treated with carboplatin and paclitaxel alone [117]. A phase 1 trial in pancreatic cancer reported an increased overall survival (12 months compared to 6 months in historical controls) and progression-free survival (26 weeks compared to 9 weeks in historical controls), indicating a potential for P-AscH- in the clinical management of pancreatic cancer, a malignancy known for poor outcomes [118]. P-AscH^−^ is currently being investigated in a phase 2 trial with gemcitabine in pancreatic cancer (NCT02905578). Other phase 1 and 1/2a trials reported similar findings in support of using P-AscH^−^ to treat pancreatic cancer [119,120]. Furthermore, in a phase 2 clinical trial on patients with advanced NSCLC, P-AscH^−^ combined with carboplatin and paclitaxel demonstrated a disease control rate of 84.2% and a confirmed objective response rate of 34.2%, relative to historical controls (15–19%) [103,121]. Interestingly, the phase 2 trial also reported an increase in effector CD8^+^ T cells demonstrated by P-AscH^−^, thus highlighting the potential of P-AscH^−^ in eliciting an anti-tumor immune response in humans [121]. The effect of P-AscH^−^ on immune cells was also reported by recent preclinical studies that combine P-AscH^−^ with immune checkpoint inhibitors (anti-PD-1 and anti-CTLA-4) in lymphoma, renal cell carcinoma, melanoma, colorectal, pancreatic, and breast cancer [122,123]. Thus, mounting evidence indicates that P-AscH^−^ acts as a radio and chemosensitizer with the potential to modulate immunotherapy responses.

In contrast to acting as a radio and chemosensitizer, data suggest that P-AscH^−^ may also exhibit protective effects in non-malignant tissues against chemotherapy and radiation. Cancer therapy-induced normal tissue toxicities constitute a significant challenge that faces cancer survivors. Conventional chemotherapeutic agents, including platinum-based drugs (cisplatin, carboplatin, etc.), are known to induce nephrotoxicity, neurotoxicity, and hepatotoxicity in pediatric and adult cancer patients [124]. Radiation also exhibits a wide array of toxic effects on normal tissues, including organ dysfunction, chronic inflammation, and fibrosis in 25% of breast cancer patients, 20% of lung cancer patients, and up to 50% of head and neck cancer patients [125]. The severe effects of chemoradiation prove dose-limiting, such that it can limit the clinical outcome and the quality of life of cancer survivors. Preclinical studies in P-AscH^−^ have highlighted its potential protective effects in combating chemoradiation toxicities. Treatment with P-AscH^−^ in rats receiving abdominal irradiation protected goblet cells in the ileum by reducing mitochondrial damage and ER degranulation [126]. Similarly, P-AscH^−^ combined with the ATM inhibitor, KU60019, significantly reduced radiation-induced intestinal injury in mice, as evidenced by significant decreases in lipid peroxidation, 3-nitrotyrosine, and TGF-ß [115]. Additionally, P-AscH^−^ protected human erythrocytes from taxol toxicity and restored their structural integrity and antioxidant function, highlighting the potential of P-AscH^−^ in mitigating the normal tissue toxicities of taxols such as paclitaxel [127]. P-AscH^−^ also reversed DNA damage, lipid peroxidation, and jejunal injury in a study combining gold–palladium–polyethylene glycol-coated (Au@Pd-PEG) nanoparticles and radiation [128]. The protective effects of P-AscH^−^ were also observed in clinical settings, wherein patients enrolled in a phase 1/2a trial of ovarian cancer reported that P-AscH^−^ significantly reduced adverse events (grade 1) associated with carboplatin and paclitaxel, while showing a trend toward substantially reducing grade 2 adverse events [117]. In a small clinical trial sample (phase 1), P-AscH^−^ exhibited potential normal tissue protection against gemcitabine and radiation, where patients treated with P-AscH^−^ showed decreased plasma levels of F2-isoprostane, a marker of oxidative stress [118,129]. Moreover, further analysis of duodenal samples and samples from pancreatic cancer patients treated with chemoradiation ± P-AscH^−^ showed the possible reversal of villi damage and lipid peroxidation [130]. 

Pre-clinical data and early-phase clinical trials show that P-AscH^−^ is an effective strategy to selectively enhance tumor control with chemoradiation. While it may appear that P-AscH^−^ is an ideal approach to improving cancer therapy outcomes, multiple questions regarding its efficacy and limitations to its clinical use need to be addressed through further investigation. For example, P-AscH^−^ combined with FOLFOX ± Bevacizumab demonstrated promising patient outcomes in a randomized phase 3 trial in treatment-naïve patients with unresectable metastatic colorectal cancer [131]. However, this effect was limited to patients with RAS mutations, as P-AscH^−^ significantly increased progression-free survival to 9.2 months vs. 7.8 months in the control group [131]. This highlights the need for addressing the role of the tumor mutational profile in colorectal cancer responses to P-AscH^−^, as it appears to be a prognostic factor for clinical outcomes. Similarly, a single-arm phase 2 trial in castration-resistant prostate cancer patients reported no significant changes in patient outcomes with the use of P-AscH^−^, despite pre-clinical studies reporting tumor control benefits in prostate cancer [101,105,132]. The lack of clinical efficacy for P-AscH- in castration-resistant prostate cancer was recently confirmed in a placebo-controlled randomized clinical trial with P-AscH^−^ and docetaxel, where P-AscH^−^ did not demonstrate any additional improvements in patient responses or toxicities [133]. In pre-clinical models of soft tissue sarcoma, P-AscH^−^ significantly enhanced the efficacy of gemcitabine and radiation therapy by promoting DNA damage [102]. Subsequently, two clinical trials were initiated to investigate P-AscH^−^ in soft tissue sarcoma. P-AscH^−^ combined with gemcitabine was investigated in a phase 2 trial (NCT03468075) that was terminated due to the lack of any patient responses. Another trial investigated P-AscH^−^ with radiation (NCT03508726) and was completed, but reports on patient outcomes are yet to be published. These studies signify the need for further investigation to address variations from preclinical to clinical responses, heterogeneous patient outcomes, and possibly ascorbate resistance. Glioblastoma makes a case for continuing research efforts with P-AscH^−^, wherein a heterogenous patient response was observed despite significant improvements in GBM patient outcomes with P-AscH^–^ [103,116,134]. Moreover, patients with tumors containing a high iron content, as revealed using iron-sensitive MRI, were significantly more responsive to P-AscH^−^ therapy [116,134]. As a result, a new clinical trial (NCT04900792) has been initiated to investigate the utility of iron oxide nanoparticle supplementation, to increase intratumoral iron to improve P-AscH^−^ responses in GBM patients [135]. Congruent to these results, recent investigations have revealed that steady-state differences in H_2_O_2_ clearance associated with GBM molecular subtypes and intratumoral iron content contribute to differential P-AscH- responses [116,134,135,136]. Therefore, concerted translational research efforts are needed that robustly contextualize and optimize the use of P-AscH^−^ across various disease sites.

**Table 2:** Summary of discussed clinical trials utilizing P-AscH^−^ and their reported outcomes.

Trial	Disease	Therapy	Outcomes	Reference
NCT02344355 Phase 2	GBM	P-AscH^−^ with radiation and temozolomide.	Increased overall survival (19.6 months) compared to 14.6 months in historical controls.	[116]
NCT00228319 Phase 1/2a	Ovarian cancer	P-AscH^−^ with carboplatin and paclitaxel.	- Increased progression-free survival (25.5 months compared to 16.75 months with carboplatin and paclitaxel alone). - Significant decrease in grade 1 adverse events.	[117]
NCT01049880 Phase 1	Pancreatic cancer	P-AscH^−^ with gemcitabine.	- 12 months overall survival (versus 6 months historical control). - 26 weeks progression-free survival (compared to 9 weeks in historical controls). - Potential decrease in oxidative damage effects and duodenal damage.	[118]
NCT00954525 Phase 1	Pancreatic cancer	P-AscH^−^ with gemcitabine and erlotinib.	P-AscH^−^ was well tolerated.	[119]
NCT01364805 Phase 1/2a	Pancreatic cancer	P-AscH^−^ with gemcitabine.	Trend toward increasing overall survival.	[120]
NCT02905578 Phase 2	Pancreatic cancer	P-AscH^−^ with gemcitabine.	Ongoing trial.	-
NCT02420314 Phase 2	NSCLC	P-AscH^−^ with carboplatin and paclitaxel.	- Disease control rate 84.2%. - Objective response rate of 34.2% compared to 15–19% in historical controls. - Increased effector CD8^+^ T cells.	[121]
NCT03146962 Phase 3	Colorectal cancer	P-AscH^−^ with FOLFOX ± Bevacizumab.	Increased progression-free survival to 9.2 months vs. 7.8 in the control group. This effect was limited to patients with RAS-mutant tumors.	[131]
NCT01080352 Phase 2	Prostate cancer	P-AscH^−^	No benefits to the use of P-AscH- as a treatment.	[132]
NCT02516670 Phase 2	Prostate cancer	P-AscH^−^ with docetaxel.	No benefits from the addition of P-AscH- to the treatment regimen.	[133]
NCT03468075 Phase 2	Soft tissue sarcoma	P-AscH^−^ with gemcitabine.	Terminated.	-
NCT03508726 Phase 1b/2	Soft tissue sarcoma	P-AscH^−^ with radiation.	Completed.	-
NCT04900792 Phase 1	GBM	P-AscH^−^ with ferumoxytol, radiation, and temozolomide.	Ongoing trial.	-

### 4.2. Superoxide Dismutase Mimetics 

Superoxide dismutase (SOD) is an ${O_{2}}^{∙-}$-scavenging enzyme that exists in several isoforms; manganese SOD in the mitochondria (SOD2/MnSOD), copper/zinc SOD in the cytosol (SOD1 or Cu/ZnSOD), and extracellular SOD (SOD3/ECSOD) [137,138]. The primary product of the reactions driven by these SOD enzymes is H_2_O_2_ (Equation (1)), making pharmaceutical mimetics of SOD enzymes valuable tools to generate therapeutic levels of H_2_O_2_ to enhance cancer cell killing.

(1) $$2O_{2}^{∙-}+2H^{+}\;\overset{SOD,\;\;\;k\;\approx \;6.4*10^{9}\;{M^{-1}s}^{-1}}{\to }\;H_{2}O_{2}+O_{2}$$

The interest in generating H_2_O_2_ using SOD mimetics dates to the 1970s, and by the 1990s, some of the earliest SOD mimetics were characterized and studied in vitro [139]. Currently, there are several classes of SOD mimetics, such as Mn salens, Mn-metalloporphyrins, Mn (II)-cyclic polyamines, and others that exhibit dismutase activity comparable to that of the native MnSOD enzyme (k_cat_ ≈ 2 × 10^9^ M^−1^s^−1^) [139,140,141,142,143]. Like P-AscH^−^, SOD mimetics yield excess amounts of H_2_O_2_ that are thought to be toxic to cancer cells but are efficiently removed by non-malignant cells. However, SOD mimetics are often more studied as protective agents in normal tissue against the toxicities of conventional cancer therapy, due to their remarkable capacity to remove toxic ${O_{2}}^{∙-}$ radicals that are generated by chemotherapy and radiation. This section focuses on the pre-clinical and clinical studies that utilize SOD mimetics to protect normal tissues from chemotherapy and radiation-induced toxicities, as well as providing recent insights into the utilization of these SOD mimetics as potential chemo and radiosensitizers. 

Mn salens are a class of bioavailable low-molecular-weight SOD mimetics. The salen complex, EUK-113 (analogs EUK-207 and EUK-189), exhibits a catalytic rate constant between 8 × 10^5^ and 1.23 × 10^6^ M^−1^s^−1^ [144,145]. EUK-207 has been reported to mitigate radiation-induced cognitive impairment in a murine model of hippocampal radiation injury [146]. In this study, the administration of EUK-207 for up to 3 months post-irradiation resulted in a significant reduction in cognitive impairment and a potential decrease in 3-NT staining in the brain [146]. Multiple studies reported similar results in radiation-induced lung injury using EUK-207 and EUK-189. Langan et al. presented preliminary evidence on EUK-189 potentially reducing DNA damage in the lung fibroblasts of partially irradiated rat lungs [147]. However, their dosing scheme failed to prevent the long-term morbidity associated with lung irradiation [147]. Later, Mahmood et al. reported that EUK-207, in combination with genistein, successfully mitigated radiation injury in rats, as evidenced by reduced fibrosis, normalized breathing rates, reduced DNA damage, TGDF-ß, and pro-inflammatory macrophages up to 7 months post-radiation, in addition to increasing survival [148,149]. These findings were consistent with another study by Gao et al., who reported a reduction of pneumonitis and fibrosis using EUK-207 in rats exposed to whole-thorax or whole-body irradiation [150]. Lastly, EUK-207 was reported to mitigate radiation-induced dermatitis and wound healing impairment in rats [151]. In this study, EUK-207 was shown to reduce dermatitis up to 90 days post-irradiation, accelerate wound healing, increase wound vasculature, and restore skin integrity and gene expression [151]. While reports on EUK-189 and EUK-207 indicated a promising potential in radioprotection, they were not explored in a clinical setting. 

Mn-metalloporphyrins are another class of SOD mimics that have been studied both as anticancer drugs and as radioprotectants for normal tissues. MnTE-2-PyP (MnE) was one of the earliest Mn-metalloporphyrins to be investigated in the context of radioprotection [139]. Although MnE effectively mitigated radiation injury in the lungs, rectum, and pelvis, the bioavailability of these drugs was limited due to their inability to cross the blood–brain barrier [152,153,154,155,156,157,158]. Two highly lipophilic analogs to MnE (MnHex and MnBuOE) were investigated in murine studies [159,160,161]. Although both drugs adequately crossed the blood–brain barrier, MnHex was associated with toxicity, thus raising concerns about long-term dosage [161]. Consequently, MnBuOE became one of the most studied bioavailable Mn-metalloporphyrins. In the field of radiation-induced brain injury, MnBuOE demonstrated the capacity to promote hippocampal neurogenesis, Dcx^+^ neuron production, restoration of corpus callosum axons, and restoration of cognitive function [161,162]. Similar effects were reported with chemotherapy-induced brain damage as MnBuOE was able to restore spatial memory and dendritic length following treatment with doxorubicin, cyclophosphamide, and paclitaxel [163]. Additionally, when combined with Carbenoxolone-Mediated TRAIL in GBM, MnBuOE enhanced tumor killing by selectively promoting apoptosis in GBM cells but not in normal astrocytes [164]. In head and neck cancer, MnBuOE efficiently reduced mucositis and fibrosis in murine models receiving radiation to the oral mucosa and salivary glands [165,166]. Contrarily, MnBuOE was able to sensitize head and neck tumor xenografts to radiation, as evidenced by increased tumor necrosis and immune infiltrates [165,166]. Similar anticancer effects of MnBuOE were also reported, in combination with radiation, in murine melanoma and mammary carcinoma, with carboplatin in ovarian cancer, and with cisplatin in NSCLC [167,168,169]. These data demonstrate the dual ability of MnBuOE to act as a radioprotective agent in normal tissues and enhance radiation efficacy in tumor tissues. These pre-clinical studies have resulted in the initiation of multiple ongoing clinical trials investigating MnBuOE in tumor control and normal tissue protection in locally advanced head and neck cancer (NCT02990468), in high-grade glioma (NCT02655601), anal cancer (NCT03386500), and rectal cancer (NCT05254327) (Table 3). 

Mn (II)-cyclic polyamines are a relatively new class of small-molecule SOD mimetics developed in the early 2000s [170]. Avasopasem manganese (also known as AVA, GC4419, or M40419) is a Mn (II)-cyclic polyamine that has recently emerged in preclinical studies and clinical trials, with characteristics that distinguish it from other SOD mimetics. With a catalytic rate constant of 2 × 10^7^ M^−1^s ^−1^, AVA is highly specific to ${O_{2}}^{∙-}$. This specificity of AVA to ${O_{2}}^{∙-}$ relies on the Mn(II) center of AVA, which does not react with other forms of ROS such as H_2_O_2_ and nitric oxide (NO) and, thus, cannot undergo the Fenton-like reactions associated with Mn(II) recycling [171,172]. This represents a fundamental divergence from the chemical activity of the Mn(II) centers of EUK-207 and MnBuOE, which were reported to have dual SOD/catalase activity, in addition to reacting with some nitrogen species, indicating the ability to undergo reversible oxidation reactions with H_2_O_2_ [144,170,173,174,175]. One of the earliest reports of AVA as a radioprotective agent was in a hamster model of radiation-induced oral mucositis, where it successfully reduced the severity and duration of oral mucositis [176]. Thompson and colleagues reported that AVA protected mice from lethal total-body irradiation (8.5 Gy) in a dose-dependent manner, as at the highest dose (40 mg kg^−1^), 100% of irradiated animals survived until day 30 post-irradiation, compared to the vehicle controls where 100% of animals died by day 17 [177]. This study also reported a marked reduction in intestinal villi loss and restored lymphoid and hematopoietic tissue integrity compared to vehicle controls [177]. In human fibroblasts, AVA was able to reverse the toxicity of radiation and chemotherapy (cisplatin and carboplatin) by reducing the aging-associated DNA damage and mitochondrial dysfunction that promote age-dependent toxicity in human dermal fibroblasts [178]. In a clinical setting, AVA has been studied as a radioprotective agent against oral mucositis in head and neck cancer patients treated with radiation and cisplatin (Table 3) [179,180,181]. AVA was reported in a phase 1/2a trial to be safe and effective at reducing the incidence of severe oral mucositis by 50% compared to historical controls [181]. A randomized double-blind phase 2b trial was initiated to follow up on these findings and reported that AVA significantly reduced the incidence of severe oral mucositis to 43%, compared to 65% in the placebo group [180]. Additionally, AVA protected against cisplatin-induced renal injury in phase 2b patients, as indicated by a reduced estimated glomerular filtration rate and epithelial growth factor [182]. These findings were later confirmed in a murine model of cisplatin-induced renal injury, where AVA was able to mitigate both chronic and acute kidney injury by restoring mitochondrial function and suppressing renal inflammation by downregulating TNFα, IL1, ICAM-1, and VCAM-1 [183]. The investigation of AVA as a therapeutic agent for oral mucositis was concluded with a phase 3 double-blind placebo-controlled trial, which showed that AVA could significantly reduce the incidence (54% compared to 64% in placebo controls) and duration (8 days compared to 18 days in placebo controls) of oral mucositis in head and neck cancer patients with radiation and cisplatin [179]. These recent studies showed that AVA is effective at enhancing radiation in lung cancer and soft tissue sarcoma and accelerating wound healing with neoadjuvant radiation [184,185,186]. Beyond these clinical outcomes with AVA, more emerging preclinical studies continue to explore AVA as an anticancer agent and a normal tissue radioprotectant. Although these data show that AVA is potentially an effective H_2_O_2_-generating agent with benefits for tumor control and normal tissue protectant, like the case of P-AscH^−^, there are mechanistic limitations that warrant continued research with these mimetics. One study reported that two SOD mimetics (MnTePyP and EUK 134) significantly reduced endothelial cell proliferation [187]. While beneficial for suppressing tumor vascularization, these data raise concerns regarding the systemic effects of excess SOD activity and their potential long-term effects on normal endothelial function, and whether tumor-targeted delivery is required for SOD mimetics. The study also reported differential effects on NF-kB activation, where it was downregulated by EUK-134 in both cancer cells and endothelial cells and upregulated in both by MnTmPyP [187]. The effect of EUK-134 is consistent with findings reported by Mapuskar et al. that TNFα, an inducer of NF-kB activation, was downregulated by AVA [183]. These variations in effects on inflammatory responses following treatment with SOD mimetics warrant further investigation as to their effects on immune cell function and inflammatory signaling. Moreover, previous studies provided evidence on aging as a potential factor that affects responses to SOD mimetics, as the SOD mimetic AVA was shown to exert protective effects in normal tissues in fibroblasts from older patients against chemoradiation, older mice against cisplatin-induced renal injury, and in older patients with cisplatin-induced acute kidney injury [178,183]. Therefore, more studies are needed to address the responses of SOD mimetics in aging, to better target the patient populations that may benefit from them in future clinical trials.

**Table 3:** Summary of discussed clinical trials utilizing SOD mimetics and their reported outcomes.

Trial	Disease	Therapy	Outcomes	Reference
NCT02990468 Phase 1/2	Head and neck cancer	MnBuOE with radiation and cisplatin	Ongoing trial.	-
NCT02655601 Phase 2	High-grade glioma	MnBuOE with radiation and temozolomide	Ongoing trial.	-
NCT03386500 Phase 1	Anal cancer	MnBuOE with radiation, 5FU, and mitomycin	Ongoing trial.	-
NCT05254327 Phase 2	Rectal cancer	MnBuOE with radiation and chemotherapy (oxaliplatin, leucovorin, fluorouracil, capecitabine)	Ongoing trial.	-
NCT01921426 Phase 1b/2a	Head and neck cancer	AVA with radiation and cisplatin	Reduction in the incidence of severe radiation-induced oral mucositis by 50% (relative to historical controls).	[183]
NCT02508389 Phase 2b	Head and neck cancer	AVA with radiation and cisplatin	- Significant reduction in severe radiation-induced oral mucositis (43% vs. 65% in the placebo control). - Reduction in estimated glomerular filtration rate, and epithelial growth factor indicating protection against cisplatin-induced renal toxicity.	[180,182]
NCT03689712 Phase 3	Head and neck cancer	AVA with radiation and cisplatin	- Significant reduction in the incidence of radiation-induced oral mucositis (54% compared to 64% in the placebo control). - Significant reduction in the duration of radiation induced oral mucositis (8 days vs. 18 days in the placebo control).	[181]

## 5. Concluding Remarks

H_2_O_2_ is a vital signaling molecule, with over 1000 target proteins that can be modified via cysteine oxidation. This cell signaling function leads to H_2_O_2_ accumulation in cancer cells, promoting tumor growth. Therefore, extensive efforts have been made to study H_2_O_2_ as a potential therapeutic target, especially given the increasing incidence of cancer, the limited treatment options for some malignancies, and the dose-limiting toxicities associated with conventional chemoradiation. H_2_O_2_-generating treatment modalities, including P-AscH^−^ and SOD mimetics, generate excess H_2_O_2_ that acts as a toxic prooxidant in cancer cells due to their reduced H_2_O_2_ clearance capacity, but as a protective antioxidant in normal cells that can efficiently remove H_2_O_2_. P-AscH^−^ was investigated in pre-clinical studies in a wide array of malignancies, including lung, ovarian, brain, skin, kidney, and colon cancer, and it was shown to exert selective anticancer effects without normal tissue toxicities. In contrast, P-AscH^−^ protected normal tissues from chemotherapy and radiation-associated toxicities. These findings were reproducible in phase 2 clinical trials in NSCLC and GBM and are currently being investigated in a phase 2 trial for pancreatic cancer. Similarly, SOD mimetics such as MnBuOE and AVA exhibited antioxidant protection in normal tissues treated with radiation and/or chemotherapy and prooxidant effects in cancerous tissues that enhanced tumor cell killing. SOD mimetics in the clinic were successful in the mitigation of severe oral mucositis in head and neck cancer patients and are actively being investigated in head and neck, glioma, anal, and rectal cancers. While both P-AscH^−^ and SOD mimetics are relatively well studied both in pre-clinical and clinical settings, further investigation is crucial due to their specified limitations. There is a critical need to improve therapeutic responses to rare, understudied malignancies such as sarcomas, clear renal cell carcinoma (ccRCC), and others. Therefore, expanding research on H_2_O_2_ metabolism, P-AscH^−^, and SOD mimetics in rare malignancies may yield significant benefits to these patient populations. Additionally, potential combinations between P-AscH^−^ and SOD mimetics present another noteworthy research direction that may hold value in overcoming resistance to conventional therapy and/or the limitations of P-AscH^−^ and SOD mimetics, with Heer et al. recently reporting that the combination of P-AscH^−^ and AVA significantly enhanced cancer cell killing in NSCLC and squamous cell carcinoma [188]. Long-term clinical outcomes, the effects of sex and age, and the feasibility of the clinical use of P-AscH^−^ and SOD mimetics are to be considered and addressed in future pre-clinical and clinical studies. Thus, exploiting this duality of H_2_O_2_-based therapies in cancer underscores its potential to harness oxidative stress to selectively target cancer cells, while simultaneously protecting non-cancerous tissue. Future research should focus on optimizing delivery mechanisms, understanding the underlying mechanisms and molecular interactions, and assessing chronic outcomes, to decipher the clinical benefits of this highly innovative therapeutic approach.

## Figures and Tables

**Table 1:** Summary of some discussed mechanisms by which H_2_O_2_ may induce cellular damage.

Damage Mechanism	Outcome	Reference
Mitochondrial Dysfunction	Decreased ATP synthesis, impaired mitochondrial function.	[9]
Fenton Chemistry	DNA, lipid, and protein damage.	[10]
Protein Modification	Irreversible protein oxidation and fragmentation, mitochondrial dysfunction, hyperactivation or inactivation of essential proteins (e.g., kinases).	[11,12,13]
